# The effect of opioids on the light-off pupillary reflex

**DOI:** 10.1186/s44158-026-00340-8

**Published:** 2026-01-16

**Authors:** Rachel Eshima McKay, Merlín D. Larson

**Affiliations:** Department of Anesthesia and Perioperative Care, University of California San Francisco (UCSF), San Francisco, CA 94143 USA

**Keywords:** Pupil, Opioids, Pupillometry, Neuromonitoring, Pupillary light reflex

## Abstract

**Objective:**

We examined the relationship between modeled opioid concentration and quantitative pupillary measures during remifentanil infusion sequences with particular attention to the “light-off” (LO) reflex.

**Methods:**

Ten volunteer subjects were recruited to undergo two 10-min remifentanil infusion protocols. Pupillary unrest in ambient light (PUAL) and LO were measured at baseline and every 2.5 min during the first 10-min infusion–25-min recovery sequence, and after a wash-out period, the Neurological Pupillary index (NPi) and LO were measured during an identical infusion–recovery sequence. We tested proportional change in each parameter from baseline as indicators of dynamic opioid effect.

**Results:**

On average, remifentanil decreased both LO dilation and PUAL by > 85%, decreased pupil diameter by > 48%, but did not significantly alter the NPi. Hypoxia occurred in 15/16 sequences. LO and PUAL both showed excellent discrimination between high-toxic versus zero-moderate opioid effect. In contrast to PUAL and LO, the scaled pupillary light reflex measurement (NPi) was not altered by opioids.

**Conclusion:**

LO and PUAL were robust indicators of opioid effect and provided equivalent estimates of respiratory depression risk in our healthy awake subjects. Compared with PUAL, LO offers the advantage of being intuitive and easily derived at the bedside without need for specialized software.

**Significance:**

Measurement of the pupillary LO reflex with a portable pupillometer provides a simple, discriminating measure of opioid effect.

**Summary statement:**

Remifentanil blocks the pupillary LO reflex.

**Supplementary Information:**

The online version contains supplementary material available at 10.1186/s44158-026-00340-8.

## Highlights

1. The effect of a toxic dose of remifentanil on the pupillary “light-off” reflex was studied in 10 volunteers.
2. Given in a dose that produced apnea, the light-off reflex was suppressed over 85% compared to baseline.
3. The pupillary light reflex was not changed by the drug.

**Supplementary Information:**

The online version contains supplementary material available at 10.1186/s44158-026-00340-8.

## Introduction

Recent studies have shown that *pupillary unrest in ambient light* (PUAL) offers promise for identifying high-risk opioid exposure in adult subjects [1]. The loss of PUAL that accompanies opioid intoxication is thought to reflect dose-dependent suppression of central inhibitory input to the Edinger–Westphal (EW) nucleus. Another pupillary function that depends on central EW modulation but has not been studied in conjunction with opioid exposure is the *light-off* (LO) reflex, the steady pupil dilation that is observed in awake subjects following an abrupt transition from light to dark environments. LO can be easily measured using a portable pupillometer that records pupil size continuously during illumination followed by a 7-s interval of total darkness. In contrast to PUAL, which requires spectral analysis (e.g., fast Fourier transform), the LO metric is intuitive and straightforward to calculate at the bedside. Whether LO provides equivalent discrimination of opioid exposure compared with PUAL remains to be determined.

Because the LO reflex is partially mediated by sympathetic activation, it may also convey complementary information. One report suggests that the LO reflex might provide a screening tool for the syndrome of cognitive motor dissociation in the neuro-intensive care unit [2].

The present study was designed to characterize the effects of opioid exposure on multiple quantitative pupillary measures in healthy volunteers. PUAL and LO responses were recorded at baseline and at 2.5-min intervals during a 10-min remifentanil infusion followed by a 25-min recovery period. In addition, we measured the Neurological Pupil index (NPi)—a composite metric quantifying the strength of the pupillary light reflex that is independent of baseline pupil size [3]. The value of NPi reflects the excitation of the EW nucleus by light. In that respect, it differs from the LO reflex that is generated by inhibition of the EW nucleus together with activation of the sympathetic dilator muscle. We hypothesized that like PUAL, the LO response would be suppressed by opioids in a dose-dependent manner and would recover rapidly in parallel with the well-defined pharmacokinetic profile of remifentanil [4]. We further hypothesized that the NPi would remain stable and unaffected by remifentanil administration.

## Methods

### Study subjects

This study was conducted following the Code of Ethics of the World Medical Association (Declaration of Helsinki) from October 15, 2022, to June 3, 2023. After receiving approval from our Institutional Review Board (Committee on Human Research, University of California, San Francisco, CA, USA (Approval No.: 21–34917)), and trial registration (Clinical Trial No. NCT05391555, date: May 20, 2022), we recruited healthy volunteers to participate in a study in which pupillary measures were taken at regular time intervals before, during, and after infusion of remifentanil. Criteria for participation included ASA 1–2 physical status, age 40–60, BMI < 35 kg/m^2^, no significant visual impairment or use of medication having autonomic activity that might affect the pupil, and no history of opioid use disorder or opioid ingestion within the prior 30-day period. We chose to study 20 infusions on 10 volunteers based upon consistent remifentanil responses to our infusion protocol in a previous study (McKay and Larson, 2021). All subjects provided written informed consent, and all studies were performed in the UCSF Department of Anesthesia Volunteer Study room, where ambient light was approximately 300 lx and environmental noise was minimal.

### Procedures

After an 8-h fast, each subject received 40-mg aprepitant by mouth, a 20-gauge intravenous catheter, 4-mg intravenous ondansetron, and an infusion of balanced salt solution at 150 cc/h. Standard monitors were utilized, including noninvasive blood pressure, SpO2, electrocardiogram, and end-tidal carbon dioxide concentration.

The volunteers were asked to undergo two 35-min remifentanil infusion–recovery sequences (10-min infusion and 25-min recovery) in which pupillary measurements were taken at baseline and every 2.5 min. Both 35-min sequences were undertaken on the same day, separated by a washout period of 30 min or more during which no pupillary measures were taken. During the first infusion–recovery sequence, PUAL and LO were measured sequentially, within < 30 s of each designated time point. The second infusion–recovery sequence involved the same infusion protocol–recovery protocol, but in this case, LO and NPi were taken sequentially, with a 30-s pause separating the two measurements. All LO and PUAL measurements were obtained using the NeurOptics PLR-3000 device, while NPi values were measured using the NeurOptics NPi-200.

### Pupillary measurements

Measurements were taken from the left eye with the non-measured right eye closed. During PUAL and LO measurements, a rubber cup covered the left eye immediately before initiating the measurement. During the NPi measurement, the measured eye was partially shielded from ambient light by a nonocclusive clip that stored the data for subsequent analysis.

To measure PUAL, we used the Neuroptics PLR–3000 that directed a soft halo of light of 410 lx through an occlusive rubber cup placed over the left eye. Illumination of the pupil is needed to initiate the characteristic fluctuations of pupillary diameter [5] that are thought to arise from intermittent inhibition of the EW nucleus. Methods of measurement and the algorithms to calculate PUAL have been reported previously [6]. PUAL values decline progressively as light intensity exceeds 500 lx or falls below 100 lx [7, 8].

A separate pupillometer (NPi–200) was used to evaluate the NPi. This instrument is used in clinical practice to evaluate the light reflex following traumatic brain injury [9, 10]. In our study, the light reflex measurements were taken before the LO measurement. The light reflex settings on the NPi–200 are set by the manufacturer and cannot be altered.

The LO reflex was measured in the left eye with the PLR 3000, while the right eye was covered by the operator’s hand. A 180-mW light stimulus (1500 lx) was directed into the measured eye for 3 s prior to the start of the recording sequence, which was triggered by a button release. Once the measurement began, the light remained on for one additional second and then was followed by a 7-s period of darkness. We noted the degree of maximum dilation in millimeter and the time to maximum dilation for each measurement. All measurements were stored for later retrieval within the pupillometer.

### Drug infusion and recovery

Following baseline measurements, the remifentanil infusion was started and maintained at 0.2 µg/kg/min for 5 min, increased to 0.3 µg/kg/min for an additional 5 min, and then was discontinued. This dosing regimen was selected to produce a predictable rise in remifentanil effect-site concentration using a simple, weight-based infusion that could be standardized across all subjects. Based on established pharmacokinetic models of remifentanil and prior human volunteer studies, an infusion of this magnitude is expected to achieve effect-site concentrations in the range of 4–6 ng/mL within approximately 10 min, a range consistently associated with profound respiratory depression and apnea in healthy adults. We chose this fixed protocol because it reliably spans the transition from subclinical to clearly toxic opioid exposure, which was necessary for eliciting measurable physiological responses for our experimental aims [4, 11]. At 10 min, the infusion was stopped, and data collection continued for an additional 25 min. Respiratory depression was defined as rapidly declining oxyhemoglobin saturation falling below 90%. When respiratory depression occurred, the volunteers were prompted to breathe, and supplemental oxygen was introduced via nasal cannula or face mask in sufficient quantity to restore SpO2 to > 94%.

Each volunteer was observed for at least 1 h after the conclusion of the last infusion. When they were able to ambulate and tolerate clear liquids by mouth without vomiting, the intravenous catheter was removed, and the volunteer was discharged into the care of a responsible adult.

### Statistical analysis

To evaluate the effect of time and modeled opioid concentration on pupillary measures, we employed random-coefficient mixed-effects models with robust standard errors during the infusion and recovery phases. In the first model sequence, proportional changes in LO, PUAL, and pupil diameter over time were included as fixed effects. In the second sequence, proportional changes in LO, pupil diameter, and NPi were included as fixed effects. To account for individual variability in the relationship between pupillary changes and fluctuating opioid concentration over time, subject-specific time slopes were modeled as random effects. The intercept was constrained to zero to reflect normalization to baseline values. We reported post hoc pairwise comparisons of differences in each modeled parameter using Wald chi-square with Bonferroni correction. This approach allowed us to assess both population-level trends and individual deviations in pupil dynamics over the infusion and recovery periods.

To assess the ability of pupillary measures to differentiate between time points with high (≥ 2.2 ng/mL) versus zero-to-moderate (< 2.2 ng/mL) modeled remifentanil concentrations, we constructed receiver operating characteristic (ROC) curves. Time points representing zero-to-moderate opioid concentration (0 and 2.5 min) were compared to those reflecting high-toxic opioid concentration (5–12.5 min) based on the distribution of estimated remifentanil effect site concentrations. Parameter values from baseline through 12.5 min were ranked and classified dichotomously according to the corresponding time juncture in the infusion sequence (< 2.2 vs. ≥ 2.2 ng/mL). We calculated the area under the ROC curve (AUROC) with 95% confidence intervals using the DeLong method. Differences in AUROC were reported, along with threshold ranges of pupillary measures predictive of high-toxic opioid concentrations at a specified interval likelihood ratio (iLR), with corresponding 95% confidence intervals.

Measures of central tendency and dispersion were reported as mean ± standard deviation for normally distributed variables and median (25th–75th percentile) for nonparametric data, including time-to-event measures. To convey precision and uncertainty, 95% confidence intervals were used for various estimates, such as regression coefficients and effect sizes, reflecting their applicability to a general population beyond our study sample. All data analysis was performed using Stata Version 18 (College Station, TX, USA).

## Results

### Demographics

Ten healthy volunteers participated in the study (age, 49 ± 6 years; weight, 66 ± 8 kg). The self-reported sex and race distribution reflected that of the local community (five females and five males: four Asian, two Latino, three White, one Black). Four participants did not complete the second infusion sequence, citing severe nausea during or following the first infusion. The final dataset therefore included 16 measurements of the LO reflex, 10 paired comparisons of PUAL and LO, and 6 paired comparisons of NPi and LO.

### The LO reflex

#### LO response at baseline

**Fig. 1:**
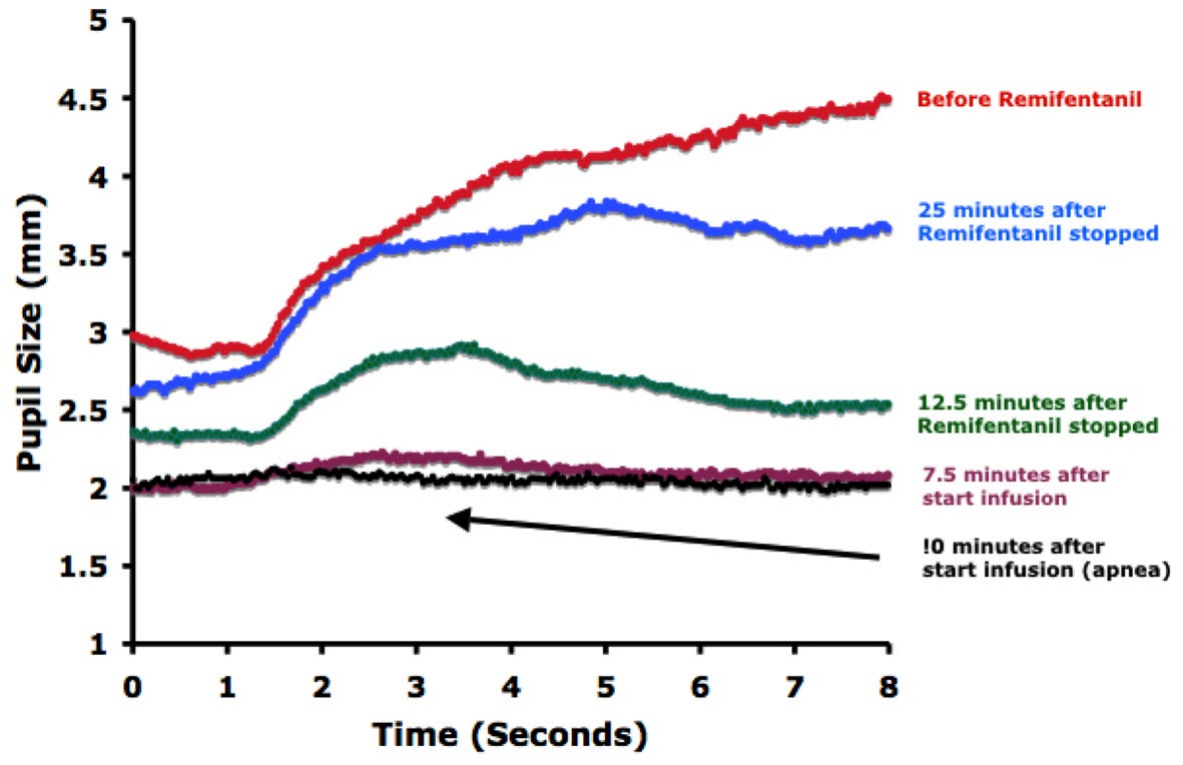
A typical remifentanil effect in one volunteer. Note the near-total ablation of the LO reflex at 10 min. This subject became apneic at the 5-min measurement time and required supplemental oxygen and prompting to breathe. Note also that the dilation is sustained before the drug is given but is not sustained during and after the infusion, declining at 3–5 s after the light went off

We analyzed 16 LO reflex trials obtained before, during, and after remifentanil infusion. At baseline, the LO response showed a progressive increase in pupil diameter throughout the 7-s dark interval, with a maximum dilation of 1.79 ± 0.47 mm occurring at a median (25th and 75th percentiles) of 7.72 (7.22, 7.99) s after light offset. A representative trace is shown in Fig. 1.

#### Clinical events during remifentanil infusion

Respiratory depression occurred in 15 of 16 trials, consistent with prior observations, at a median (25th, 75th percentile) of 5.50 (4.42, 7.15) min from infusion onset. In 13 of 15 events, verbal prompting was sufficient to reinitiate breathing during brief apnea, whereas 2 of 15 required firm mandibular support. All participants remained responsive to verbal or tactile stimulation throughout the protocol.

#### LO response at maximum opioid effect

**Fig. 2:**
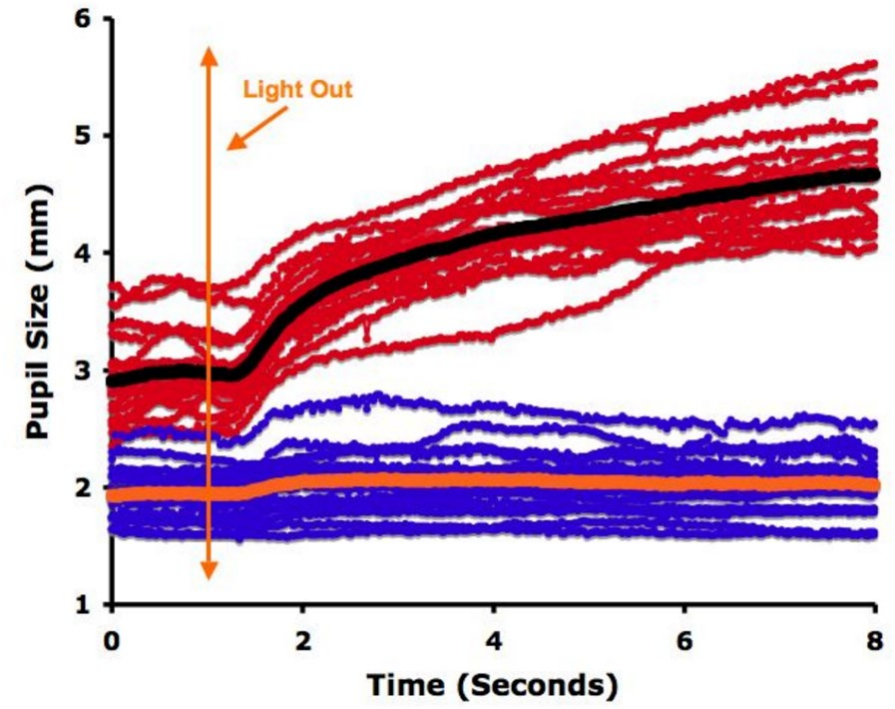
Individual pupillary tracings in 16 remifentanil infusion sequences. The LO responses in red were taken prior to drug administration. The average of the red scans is shown in black. The LO reflexes in blue are those taken 10 min after the start of the infusion. The average of the blue scans is shown in orange. Time to reach maximum pupil diameter during the 8-s measurement (median [IQR] [37]d) was significantly longer in baseline versus 10-min observations, occurring at 7.84 (7.15, 7.99) versus 3.06 (1.90, 5.62) s during the first sequence (*p* < 0.0001) and at 7.70 (7.29, 7.82) versus 3.58 (2.44, 7.25) s during the second sequence (*p* < 0.0001)

**Fig. 3:**
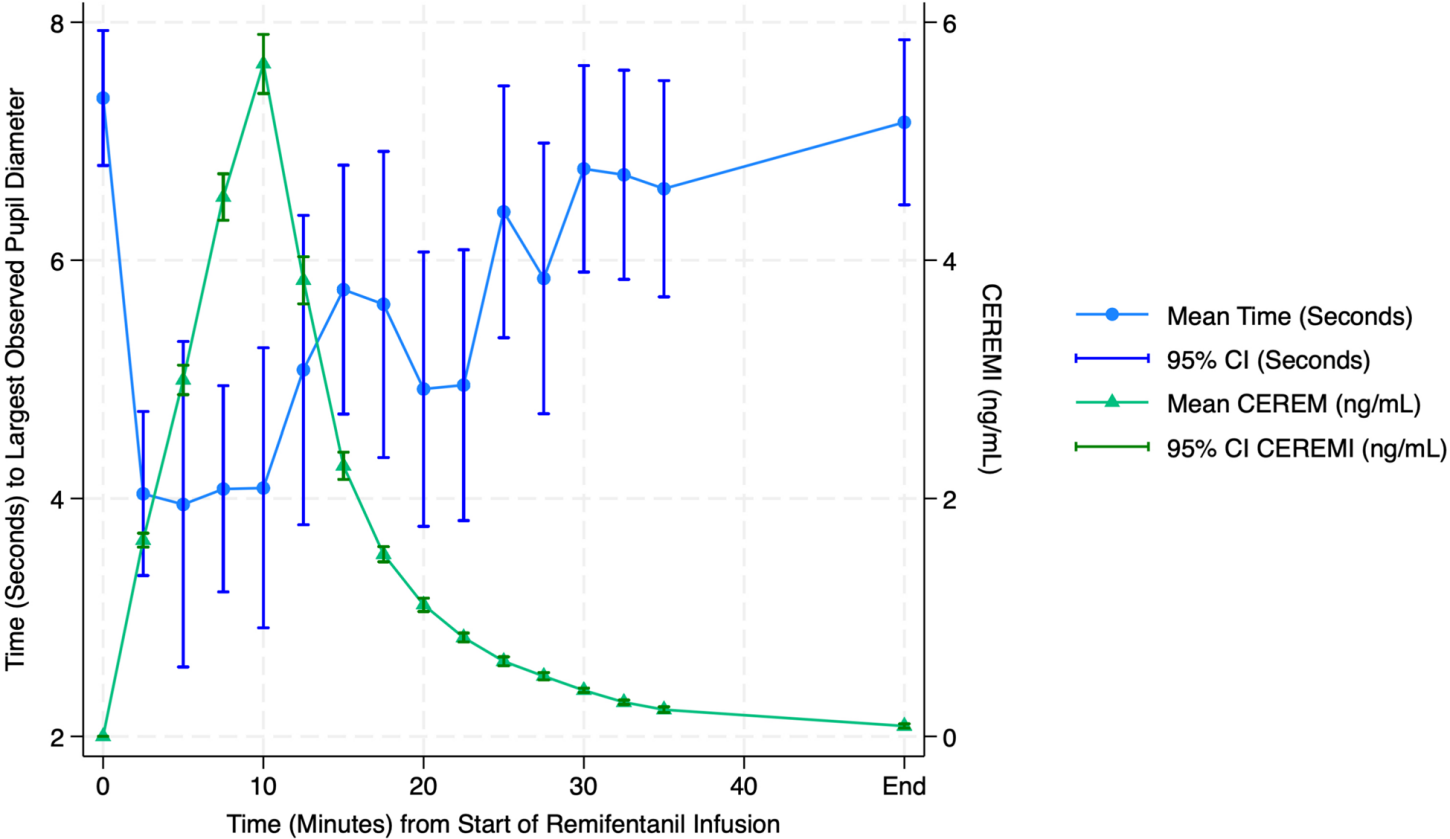
The time to maximum dilation following light off is shortened as remifentanil concentrations increase. A mixed effects model using the subject as random intercept demonstrated that CEREMI was significantly associated with timing of maximum pupil dilation (beta = −0.55 ± 0.08 s per ng/mL, *p* <.001). We conclude that the variability in time to maximum diameter was predominantly within subject rather than between subjects

**Fig. 4:**
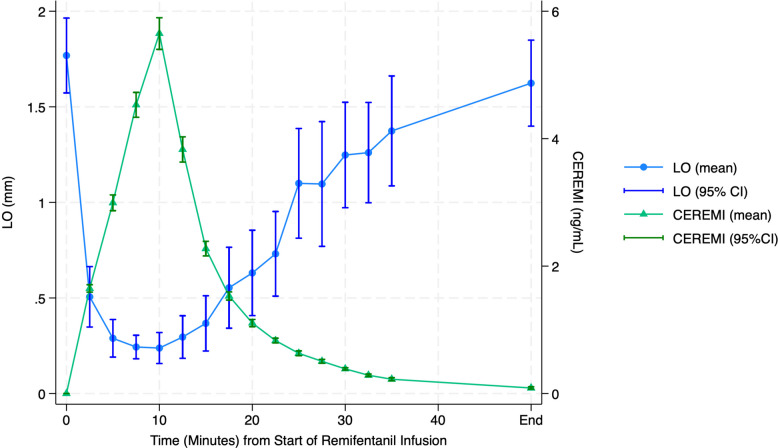
The relationship between maximum LO dilation and remifentanil concentration is shown in this figure. See Table 1 for statistical analysis

At peak remifentanil effect, maximum pupil dilation was markedly reduced: < 0.15 mm in 5, 0.15– < 0.20 mm in 3, and 0.20–0.54 mm in 8 of 16 trials. In contrast to baseline recordings, dilation during opioid effect was transient after light offset, returning toward baseline within the 8-s measurement window (Figs. 1, 2). The time to peak dilation was progressively diminished by remifentanil (Figs. 1, 2, 3). Similarly, the peak LO dilation was diminished proportionally with increasing remifentanil effect site concentrations (Fig. 4).

**Table 1:** Changes in PUAL and LO across time points

First sequence	Time (minutes) from start of 10-min remifentanil infusion				
Parameter mean (SD)	Pre (0)	10	20	30	35
Effect site remifentanil (ng/mL)	0.0	5.7 (0.5)	1.1 (0.1)	0.4 (0.1)	0.2 (0.1)
LO (mm)	1.79 (0.47)	0.22 (0.16)*	0.57 (0.36)*	1.25 (0.45)*	1.45 (0.55)*
LO % change		−86.42 (12.38)	−66.04 (21.96)	−27.43 (28.39)	−18.88 (24.91)
PUAL	0.252 (0.130)	0.025 (0.021)*	0.052 (0.040)*	0.112 (0.059)	0.162 (0.080)
PUAL % change		−87.59 (14.72)	−80.40 (9.30)	−53.05 (22.16)	−33.03 (18.53)
Diameter (mm)	4.02 (0.51)	2.04 (0.23)*	2.62 (0.41)*	3.21 (0.53)*	3.42 (0.49)
Diameter % change		−48.83 (5.95)	−34.41 (9.83)	−19.29 (13.96)	−13.92 (14.13)

Values differing vs baseline. *p < 0.001. Values differing by parameter. Maximum drug effect: % change PUAL–LO: difference = −1.16 (*p* = 1.000). Maximum drug effect: % change diameter–LO: difference = 37.59 (*p* < 0.001). Maximum drug effect: % change diameter–PUAL: difference = 38.75 (*p* < 0.001)

### Comparison of parameter changes during the first infusion sequence

**Fig. 5:**
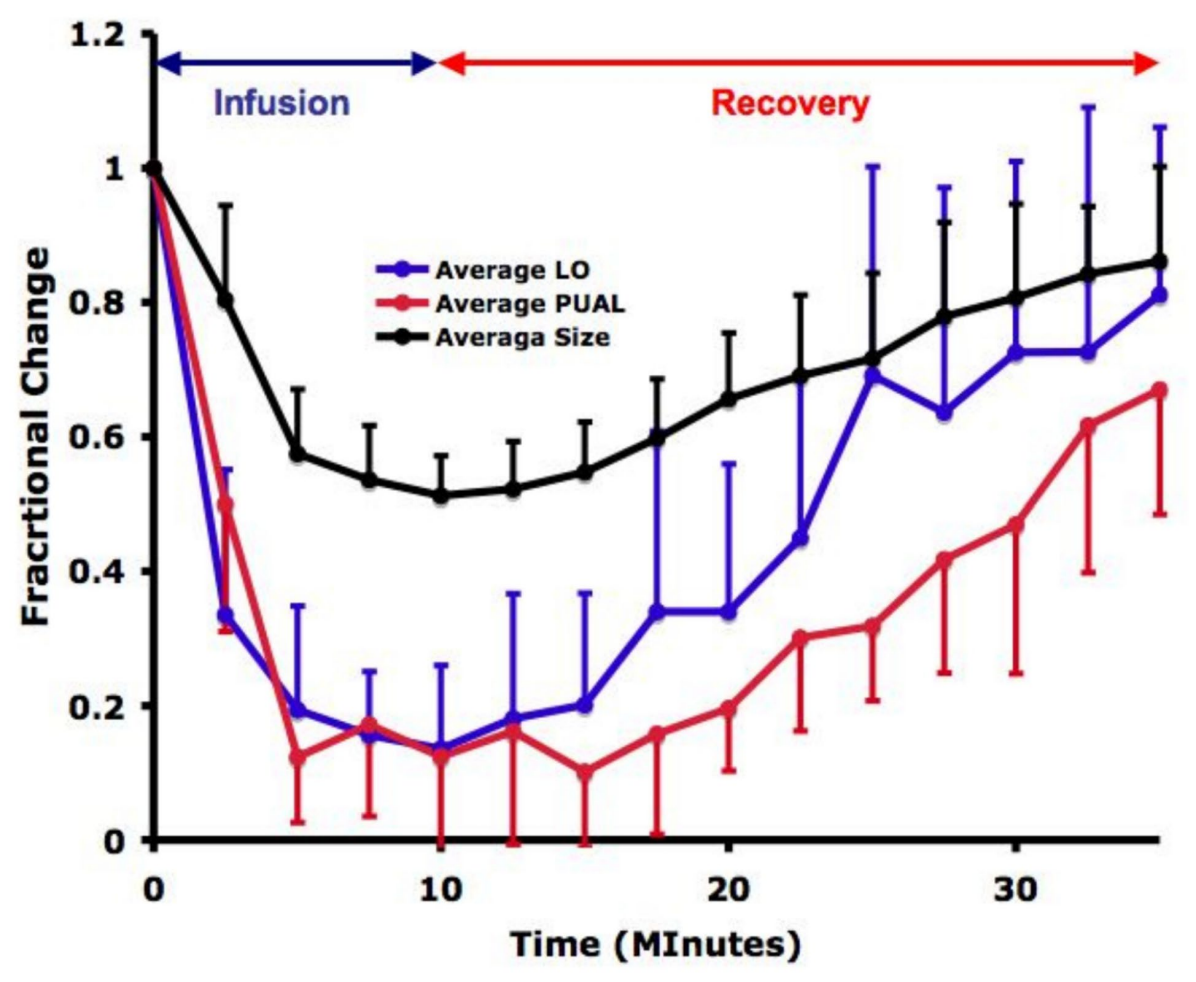
The proportional decline in LO and PUAL during remifentanil infusion (0–10 min) and recovery (10 min) did not differ significantly. Proportional change in diameter during both periods was significantly smaller than that of LO or PUAL (see Supplemental File No. 1 for statistical analysis)

Changes in PUAL and LO across time points are summarized in Fig. 5 and Table 1. The column labeled “10 min” represents the time at which the remifentanil infusion was discontinued.

Pupillary measurements at peak opioid effect differed significantly from baseline for LO, PUAL, and pupil diameter (*p* = 0.0020). The magnitude of change varied among parameters (*p* = 0.0001), with LO and PUAL exhibiting significantly greater reductions than pupil diameter (*p* = 0.0002 and *p* = 0.0001, respectively). No significant difference was observed between LO and PUAL (*p* = 0.3516).

Results from the mixed-effects models evaluating proportional pupillary parameter changes over time during infusion and recovery are provided in Supplementary File 1. A significant correlation was observed between changes in all parameters and sequential time points throughout the first infusion–recovery sequence. The standard deviation of subject-specific random slopes was significant for LO and pupil diameter during the recovery phase only, suggesting greater between-subject variability as opioid concentrations declined. Overall, the relatively small standard deviation of the random coefficients, compared with the residual standard deviation, indicated that between-subject variability accounted for only a minor proportion of total model variance.

### Analysis of the second infusion sequence

**Fig. 6:**
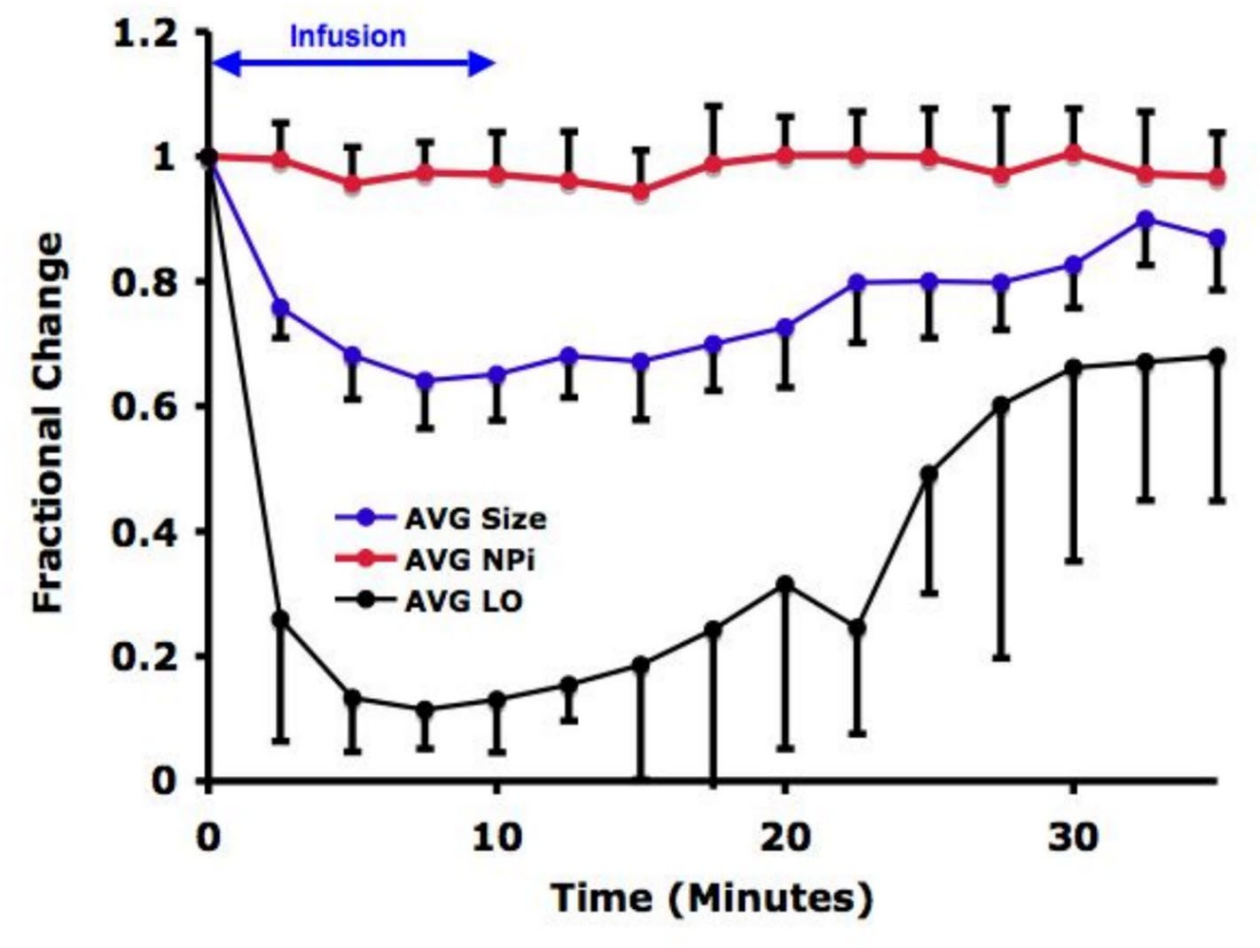
During the second sequence, the LO reflex was depressed by remifentanil to a greater extent than pupil size, but NPi values showed no significant change over time. Means ± SD. See Supplemental File 2 for statistical analysis

Four participants did not complete the second infusion sequence because of nausea. To assess whether this attrition introduced bias, we fit linear mixed-effects models with subject as a random intercept and infusion sequence (run 1 vs run 2) as a fixed effect. Infusion sequence was not a significant predictor of the outcome (Wald *χ*^2^ (1) = 2.37, *p* = 0.12), indicating that LO response across time did not differ systematically between sequences. The estimated effect of remifentanil concentration remained stable and highly significant across models. These findings suggest that discontinuation before the second sequence did not materially influence the dose-response relationships reported. In the second sequence, statistically significant changes in LO and pupil diameter over time were observed, whereas NPi values did not change significantly (*p* = 0.2905, Fig. 6). The magnitude of change differed among parameters (*p* = 0.0005), with LO demonstrating a greater decline than pupil diameter (*p* = 0.0258) and NPi (*p* < 0.0001). Inspection of Figs. 5 and 6 confirms that the changes in pupil size and LO were similar in both infusions.

### ROC analysis comparing the LO reflex to PUAL as a measure of respiratory depression

LO demonstrated excellent discrimination between high-toxic and zero-to-moderate opioid effect, with an AUROC (95% CI) of 0.8925 (0.7908, 0.9942).

LO values < 0.20 mm and > 0.70 mm were highly predictive, yielding interval likelihood ratios (iLRs) of 13.50 (1.92, 94.92) and 0.02 (0.00, 0.17), respectively, for high-toxic versus zero-moderate opioid effect. LO values between 0.200 and 0.550 mm were indeterminate (*iLR* = 1.65 [0.94, 2.91]). By comparison, PUAL also showed excellent discrimination in the same 10 sequences, with an AUROC of 0.9700 (0.9340, 1.000**)**. PUAL values < 0.06 and > 0.13 yielded iLRs of 17.95 (2.65, 121.61) and 0.00 (0.00, 0.55), respectively, for high-toxic and zero-moderate opioid effect.

## Discussion

This study demonstrates that the LO reflex, an easily measured index of Edinger–Westphal and sympathetic activity, declines in a dose-dependent fashion during opioid exposure and is nearly abolished at remifentanil concentrations producing apnea. These findings extend prior observations of opioid-induced miosis in the dark-adapted eye [12–15] by showing that the dynamic LO response, recorded over a 7-s interval following light offset, can track opioid effect in real time.

### Comparison of LO reflex to PUAL

The LO reflex and PUAL exhibited parallel proportional decline from baseline to maximum opioid effect. LO provided a robust, incremental marker of opioid exposure but with the practical advantage of requiring no spectral analysis or specialized software. The close correspondence between LO and PUAL responses suggests that the loss of LO reflex isolates the same inhibitory pathway within the Edinger–Westphal nucleus that underlies PUAL suppression.

A recent study of unmedicated ambulatory participants showed that baseline PUAL is reduced in older adults and in individuals with comorbidities known to impair autonomic regulation [16]. Whether similar influences of age and autonomic impairment extend to the LO reflex remains unknown. Environmental or sensory factors may differentially affect the two measures: pain or noise do not appear to alter PUAL within the same subject [17], but a brief noxious stimulus can transiently augment the LO response [18]. Extraneous light during the dark interval or background distractions could also modulate LO values, and these effects warrant further study.

### Comparison of LO reflex to the pupillary light reflexes as measured by the NPi

In contrast to LO and PUAL, the pupillary light reflex as quantified by the Neurological Pupil index (NPi) was not depressed by opioids. Both LO and PUAL reached near-zero values at concentrations producing opioid-induced respiratory depression. These findings confirm that the NPi metric, which is normalized for resting pupil size, is largely insensitive to opioid effects within ranges used in the study.

### Mechanism of LO depression by opioids

When light is abruptly withdrawn, the retinal ganglion cells activate inhibitory neurons that depress the Edinger–Westphal neurons. The light-off reflex also involves activation of the preganglionic sympathetic fibers that innervate the dilator muscle of the iris [19]. Although the precise sources of inhibition that contribute to PUAL and the LO reflex are unknown, various nuclei in the brain stem, including the locus coeruleus, the pedunculopontine tegmental nucleus, and portions of the raphe nucleus, are thought to be involved [19–23].

Following the administration of remifentanil, the pupil often failed to continue a steady increase in size as was observed prior to the drug. Thus, we observed a progressive decrease in the time to peak dilation as the drug effects increased. A similar effect of opioids on pupillary reflex dilation has been reported [24]. We interpret this alteration in the time to peak dilation as a progressive decrease in the duration of the spike train initiated by the LO response. Similar curtailments in the duration of neuronal responses have been observed following recordings of neuronal responses following noxious stimulation [25].

The effect of remifentanil on the sympathetic responses following LO is unclear. Because the LO reflex was nearly eliminated in many of the cases, we think that the drug also depressed the sympathetic excitation of the dilator muscle that normally occurs following LO. It is known that opioids depressed the functional activity of neurons in the locus coeruleus [26, 27]. This nucleus activates the sympathetic dilator muscle and indirectly blocks inhibition at the Edinger–Westphal nucleus [28–31]. It is possible that the effects on the LO reflex by remifentanil are brought about by a depressant action within the locus coeruleus.

The LO reflex should not be confused with pupillary reflex dilation (PRD). PRD is generated by an arousing stimulus [32] and is primarily mediated by activation of the dilator muscle in awake subjects and by inhibition of the EW nucleus during general anesthesia [33]. Opioids block the dilation initiated by PRD [34, 35] and by LO.

### Limitations

We did not study subjects with age > 60 or with comorbid conditions associated with possible impairment of autonomic function, such as diabetes or cardiovascular disease requiring treatment with beta-blockers. Further investigations in these populations would be useful. Because our contention that the opioid induced depression of the LO reflex involves a similar mechanism to the depression of PUAL, we would expect advanced age would have the same effect on both responses.

The remifentanil infusion rates were intentionally selected to achieve a range of opioid concentrations, from subclinical-moderate to clearly toxic, within a 10-min window. This approach characterized the dose-response relationship between opioid exposure and pupillary dynamics. The rapid remifentanil escalation was not designed to mimic typical clinical opioid administration, where effect-site concentrations rise more gradually and responses may be influenced by patient comorbidities and concomitant use of centrally acting medications. Accordingly, the concentrations achieved in this experimental paradigm should not be interpreted as directly generalizable to all clinical populations or practice settings; rather, they provide controlled, mechanistic insight into opioid-induced respiratory and pupillary responses under tightly standardized conditions.

### Clinical application

The LO reflex can be readily retrieved from portable pupillometers and shows a robust, statistically significant inverse relationship to modeled opioid concentration. This opioid-induced pupillary relationship was highly consistent between individual subjects. Specified ranges of LO reflex values show excellent discrimination between high-toxic versus zero-moderate opioid concentrations.

A recent study of comatose patients demonstrated that nearly 25% were intermittently awake, with evidence of cognitive motor dissociation [36]. One application of the LO reflex would be a simple screening test to detect the awake state in patients with cognitive-motor dissociation following brain injury [2]. Based on findings from their study, an abnormally low or absent LO reflex in a critically ill patient may indicate a lack of consciousness, but, as the present report has demonstrated, attention to the concomitant use of opioids should be considered.

## Conclusions

Under our experimental conditions, we observed that at peak remifentanil concentration, the LO reflex was either completely abolished or showed only a brief dilation followed by gradual constriction. Notably, the LO reflex recovered rapidly after remifentanil was discontinued. The magnitude of both the LO reflex and PUAL decline appeared comparable as opioid concentrations rose. In contrast, the Neurological Pupil index (NPi) remained stable even during remifentanil-induced apnea.

## Supplementary Information

Supplementary Material 1. Results from the mixed-effects models.

Supplementary Material 2. Statistical analysis.

## Data Availability

Research data in this submission that is not included in the Results section, will be made available on request.
