# Supplementary material for: The effect of opioids on the light-off pupillary reflex
Source: J Anesth Analg Crit Care. 2026 Jan 16;6:25. doi: 10.1186/s44158-026-00340-8 (PMC12892534; doi:10.1186/s44158-026-00340-8)
Supplement: Supplementary file 1 — Supplementary Material 1. Results from the mixed-effects models. [file 44158_2026_340_MOESM1_ESM.docx]

Supplementary File 1:

| Sequence 1: | Random Slope Model: % Parameter Change per Minute | | |
| --- | --- | --- | --- |
| Infusion (0-10 min) | Light Off (LO) | PUAL | Pupil Size (Diam) |
| Fixed Effects |  |  |  |
| Coefficient | -7.628^***^ | - 8.322^***^ | - 4.980^***^ |
| Robust SE | (0.246) | (0.367) | (0.114) |
| Random Effects |  |  |  |
| SD coefficient | 0.0000106 | 0.0000897 | 0.705846 |
| SD residual | 22.41246 | 20.88129 | 9.89835 |
| Chi2 (p-value) for Random Coefficient | -0.00(1.0000) | -0.00 (1.0000) | 2.03 (0.1542) |
| Pair-Wise Comparisons with Bonferroni Correction | | | |
|  | Contrast | Robust SE | P-value |
| PUAL vs LO | 1.96 | 3.67 | 1.000 |
| Diam vs LO | 32.08^***^ | 3.25 | <0.001 |
| Diam vs PUAL | 30.13^***^ | 2.17 | <0.001 |
| *N* | 50 | 50 | 50 |

Standard errors in parentheses

^*^ *p* < 0.05, ^**^ *p* < 0.01, ^***^ *p* < 0.001

During the first sequence, the magnitude of change over time was significantly greater for Light Off and PUAL compared to Pupil Size during the 10-minute remifentanil infusion (p < 0.001). The magnitude of Light Off versus PUAL change over these time-points did not differ significantly (p = 1.000). The standard deviation for the random slope over time did not reach statistical significance for any of the three parameters, indicating no substantial contribution of between-subject variability in the rate of parameter change over time during infusion.

| Sequence 1: | Random Slope Model: % Parameter Change per Minute | | |
| --- | --- | --- | --- |
| Recovery (10-35 min) | Light Off (LO) | PUAL | Pupil Size (Diam) |
| Fixed Effects |  |  |  |
| Coefficient | 2.832^***^ | 2.288^***^ | 1.538^***^ |
| Robust SE | (0.303) | (0.337) | (0.166) |
| Random Effects |  |  |  |
| SD coefficient | 0.8975 | 0.1864 | 0.3676 |
| SD residual | 14.7608 | 7.0151 | 5.6654 |
| Chi2 (p-value) for Random Coefficient | 92.72 (<0.0001) | 2.17 (0.1409) | 101.37 (< 0.0001) |
| Pair-Wise Comparisons with Bonferroni Correction | | | |
|  | Contrast | Robust SE | p-value |
| PUAL vs LO | -14.8193 | 7.2833 | 0.126 |
| Diam vs LO | 21.5073^**^ | 6.1259 | 0.001 |
| Diam vs PUAL | 36.3266^***^ | 2.7709 | <0.001 |
| *N* | 110 | 110 | 110 |

Standard errors in parentheses

^*^ *p* < 0.05, ^**^ *p* < 0.01, ^***^ *p* < 0.001

During the recovery phase of the first sequence, the magnitude of change over time during recovery was significantly greater for Light Off (P = 0.001) and PUAL (P < 0.001) compared to Pupil Size (P < 0.001). The difference in magnitude of change the same time-points for Light Off and PUAL did not differ statistically when robust standard errors were used (P = 0.126).

The standard deviation for the random slope over time was statistically significant for LO and Pupil Size, indicating between-subject variability in the rate of parameter change over time during recovery. However, compared to the residual variance, the random slope standard deviation was small, indicating that between-subject variability accounted for a very small proportion of the total model variability.

**Comparisons of proportional pupillary parameter change during the first infusion-recovery sequence:**

| **Time (min) from start of infusion** | **LO** | **PUAL** | **Pupil Size** | **KW Chi2**  **(p-value)** | **Pairwise Comparisons**  **(Dunn Test)** |
| --- | --- | --- | --- | --- | --- |
|  | **Mean (95% CI)** | | |  |  |
| **2.5** | **-66.51**  **(-81.93, -51.08)** | **-50.01**  **(-63.58, -36.43)** | **-19.64**  **(-29.72, -9.57)** | **17.043**  **(0.0002)** | **PUAL vs LO (0.1213)**  **Diam vs LO (<0.0001)**  **Diam vs PUAL (0.0022)** |
| **5** | **-80.66**  **(-91.75, -69.57)** | **-87.67**  **(-94.62, -80.73)** | **-42.58**  **(-49.47, -35.68)** | **19.86**  **(0.0001)** | **PUAL vs LO (0.2385)**  **Diam vs LO (0.0003)**  **Diam vs PUAL (<0.0001)** |
| **7.5** | **-84.35**  **(-91.08, -77.63)** | **-82.89**  **(-92.65, -73.14)** | **-46.47**  **(-52.26, -40.67)** | **18.227**  **(0.0001)** | **PUAL vs LO (0.4495)**  **Diam vs LO (0.0001)**  **Diam vs PUAL (0.0001)** |
| **10** | **-86.42**  **(-95.28, -77.57)** | **-87.59**  **(-98.12, -77.05)** | **-48.84**  **(-53.09, -44.57)** | **18.356 (0.0001)** | **PUAL vs LO (0.3516)**  **Diam vs LO (0.0002)**  **Diam vs PUAL (0.0001)** |
| **12.5** | **-81.98**  **(-95.29, -68.66)** | **-83.83**  **(-95.90, -71.77)** | **-47.83**  **(-52.91, -42.74)** | **14.317**  **(0.0008)** | **PUAL vs LO (0.4797)**  **Diam vs LO (0.0006)**  **Diam vs PUAL (0.0005)** |
| **15** | **-79.90**  **(-91.80, -67.99)** | **-89.83**  **(-97.93, -81.74)** | **-45.30**  **(-50.64, -39.96)** | **19.582**  **(0.0001)** | **PUAL vs LO (0.0933)**  **Diam vs LO (0.0014)**  **Diam vs PUAL (<0.0001)** |
| **17.5** | **-66.04**  **(-85.27, -46.82)** | **-84.22**  **(-94.93, -73.51)** | **-40.25**  **(-46.57, -33.93)** | **15.920**  **(0.0003)** | **PUAL vs LO (0.0301)**  **Diam vs LO (0.0175)**  **Diam vs PUAL (<0.0001)** |
| **20** | **-66.04**  **(-81.75, -50.33)** | **-80.40**  **(-87.05, -73.74)** | **-34.41**  **(-41.44, -27.37)** | **16.880**  **(0.0002)** | **PUAL vs LO (0.1066)**  **Diam vs LO (0.0028)**  **Diam vs PUAL (<0.0001)** |
| **22.5** | **-55.02**  **(-72.36, -37.68)** | **-69.87**  **(-79.80, -59.94)** | **-30.94**  **(-39.53, -22.34)** | **102.814**  **(0.0001)** | **PUAL vs LO (0.3516)**  **Diam vs LO (0.0002)**  **Diam vs PUAL (0.0001)** |
| **25** | **-30.86**  **(-53.11, -8.62)** | **-68.16**  **(-76.11, -60.20)** | **-28.35**  **(-37.42, -19.27)** | **14.836**  **(0.0006)** | **PUAL vs LO (0.0010)**  **Diam vs LO (0.0002)**  **Diam vs PUAL (0.0001)** |
| **27.5** | **-36.38**  **(-60.28, -12.48)** | **-58.28**  **(-70.36, -46.20)** | **-22.13**  **(-32.13, -12.13)** | **11.272**  **(0.0036)** | **PUAL vs LO (0.0337)**  **Diam vs LO (0.0638)**  **Diam vs PUAL (0.0004)** |
| **30** | **-27.43**  **(-47.75, -7.12)** | **-53.05**  **(-68.91, -37.20)** | **-19.29**  **(-29.27, -9.31)** | **9.177**  **(0.0102)** | **PUAL vs LO (0.0301)**  **Diam vs LO (0.1319)**  **Diam vs PUAL (0.0014)** |
| **32.5** | **-34.66**  **(-64.21, -5.10)** | **-38.35**  **(-54.01, -22.69)** | **-15.78**  **(-22.93, -8.63)** | **6.397**  **(0.0408)** | **PUAL vs LO (0.3706)**  **Diam vs LO (0.0224)**  **Diam vs PUAL (0.0097)** |
| **35** | **-18.88**  **(-36.70, 1.07)** | **-33.03**  **(-46.28, -19.78)** | **-13.93**  **(-24.04, -3.82)** | **3.920**  **(0.1409)** | **PUAL vs LO (0.0891)**  **Diam vs LO (0.2795)**  **Diam vs PUAL (0.0268)** |
