# Supplementary material for: The effect of opioids on the light-off pupillary reflex
Source: J Anesth Analg Crit Care. 2026 Jan 16;6:25. doi: 10.1186/s44158-026-00340-8 (PMC12892534; doi:10.1186/s44158-026-00340-8)
Supplement: Supplementary file 2 — Supplementary Material 2. Statistical analysis. [file 44158_2026_340_MOESM2_ESM.docx]

Supplementary File 2:

| Sequence 2: | Random Slope Model: % Parameter Change per Minute | | |
| --- | --- | --- | --- |
| Infusion (0-10 min) | Light Off (LO) | Pupil Size (Diam) | Light Reflex (NPi) |
| Fixed Effects |  |  |  |
| Coefficient | -7.317^***^ | -3.142^***^ | -0.510 |
| Robust SE | (0.307) | (0.393) | (0.279) |
| Random Effects |  |  |  |
| SD coefficient | 0.0000206 | 1.0046 | 0.6035 |
| SD residual | 23.1827 | 7.9184 | 3.2684 |
| Chi2 (p-value) for Random Coefficient | -0.00 (1.0000) | 5.82 (0.0158) | 12.71 (0.0004) |
| Pair-Wise Comparisons with Bonferroni Correction | | | |
|  | Contrast | Robust SE | P-value |
| Diam vs LO | 41.9781^***^ | 3.7671 | <0.001 |
| NPi vs LO | 64.0892^***^ | 3.0237 | <0.001 |
| NPi vs Diam | 22.1111^***^ | 4.3216 | <0.001 |
| *N* | 30 | 30 | 30 |

Standard errors in parentheses

^*^ *p* < 0.05, ^**^ *p* < 0.01, ^***^ *p* < 0.001

During the infusion phase of the second sequence, LO and Pupil Size declined significantly over time (p < 0.001) but NPi did not change significantly (p = 0.265). The magnitude of change over time was significantly greater for Light Off versus Diameter (p < 0.001). The standard deviation for the random slope over time was statistically significant for both Pupil Size and NPi, indicating significant between-subject variability in parameter change over time during recovery. However, compared to the residual variance, the random slope standard deviation was small, indicating that between-subject variability accounted for small proportion of the total model variability.

| Sequence 2: | Random Slope Model: % Parameter Change per Minute | | |
| --- | --- | --- | --- |
| Recovery (10-35 min) | Light Off (LO) | Pupil Size (Diam) | Light Reflex (NPi) |
| Fixed Effects |  |  |  |
| Coefficient | 2.563^***^ | 1.391^***^ | 0.061 |
| Robust SE | (0.646) | (0.336) | (0.122) |
| Random Effects |  |  |  |
| SD coefficient | 0.8776 | 0.1493 | 0.2339 |
| SD residual | 16.9201 | 9.5053 | 4.9884 |
| Chi2 (p-value) for Random Coefficient | 43.98 (< 0.0001) | 3.06 (0.0800) | 37.39 (< 0.0001) |
| Pair-Wise Comparisons with Bonferroni Correction | | | |
|  | Contrast | Robust SE | P-value |
| Diam vs LO | 35.3820^***^ | 9.3451 | <0.001 |
| NPi vs LO | 52.3185^***^ | 9.7602 | <0.001 |
| NPi vs Diam | 16.9364^***^ | 3.7288 | <0.001 |
| *N* | 66 | 66 | 66 |

Standard errors in parentheses

^*^ *p* < 0.05, ^**^ *p* < 0.01, ^***^ *p* < 0.001

During the recovery phase of the second sequence, LO and Pupil Size increased significantly over time (p < 0.001) but NPi did not change significantly (p = 0.614). The magnitude of change over time was significantly greater for Light Off versus Diameter (p < 0.001). The standard deviation for the random slope over time was statistically significant for LO and NPi, indicating significant between-subject variability in the rate of parameter change over time during recovery. However, compared to the residual standard deviation, the random slope standard deviation indicates that the between-subject variability accounted for a very small proportion of the total model variability.

**Comparisons of proportional pupillary parameter change during the second infusion-recovery sequence:**

| **Time (min) from start of infusion** | **LO** | **Diam** | **NPi** | **KW Chi2**  **(p-value)** | **Pairwise Comparisons** |
| --- | --- | --- | --- | --- | --- |
| **Mean (95% CI)** | | | |  | |
| **2.5** | **-73.82**  **(-94.26, -53.39)** | **-22.33**  **(-30.78, -13.87)** | **2.42**  **(-6.76, 5.70)** | **15.158**  **(0.0005)** | **Diam vs LO (0.0250)**  **NPi vs LO (<0.0001)**  **NPi vs Diam (0.0258)** |
| **5** | **-84.75**  **(-93.81, -75.69)** | **-30.63**  **(-40.37, -20.90)** | **-4.36**  **(-10.50, 1.78)** | **15.158**  **(0.0005)** | **Diam vs LO (0.0258)**  **NPi vs LO (<0.0001)**  **NPi vs Diam (0.0258)** |
| **7.5** | **-87.61**  **(-93.35, -81.88)** | **-34.93**  **(-44.41, -25.45)** | **-2.54**  **(-7.57, 2.49)** | **15.174**  **(0.0005)** | **Diam vs LO (0.0257)**  **NPi vs LO (<0.0001)**  **NPi vs Diam (0.0257)** |
| **10** | **-84.57**  **(-92.67, -76.47)** | **-32.97**  **(-44.65, -21.29)** | **-2.88**  **(-9.95, 4.20)** | **15.158**  **(0.0005)** | **Diam vs LO (0.0258)**  **NPi vs LO (<0.0001)**  **NPi vs Diam (0.0258)** |
| **12.5** | **-81.30**  **(-90.45, -72.14)** | **-35.31**  **(-43.08, -27.53)** | **-3.90**  **(-12.19, 4.40)** | **15.158**  **(0.0005)** | **Diam vs LO (0.0258)**  **NPi vs LO (<0.0001)**  **NPi vs Diam (0.0258)** |
| **15** | **-74.25**  **(-97.12, -51.38)** | **-29.84**  **(-40.17, -19.51)** | **-5.57**  **(-12.50, 1.36)** | **15.174**  **(0.0005)** | **Diam vs LO (0.0257)**  **NPi vs LO (<0.0001)**  **NPi vs Diam (0.0257)** |
| **17.5** | **-68.63**  **(-98.20, -39.05)** | **-30.64**  **(-38.46, -22.83)** | **-1.19**  **(-10.88, 8.51)** | **13.053**  **(0.0015)** | **Diam vs LO (0.0971)**  **NPi vs LO (0.0002)**  **NPi vs Diam (0.0115)** |
| **20** | **-56.96**  **(-91.22, -22.69)** | **-24.84**  **(-35.93, -13.75)** | **0.19**  **(-6.34, 6.71)** | **12.341**  **(0.0021)** | **Diam vs LO (0.1649)**  **NPi vs LO (0.0003)**  **NPi vs Diam (0.0074)** |
| **22.5** | **-61.96**  **(-90.96, -32.97)** | **-15.68**  **(-24.36, -7.01)** | **0.24**  **(-7.02, 7.50)** | **125.222**  **(0.0001)** | **Diam vs LO (<0.0001)**  **NPi vs LO (<0.0001)**  **NPi vs Diam (<0.0001)** |
| **25** | **-47.11**  **(-72.89, -21.33)** | **-11.02**  **(-24.51, 2.47)** | **-0.12**  **(-8.24, 8.00)** | **11.099**  **(0.0039)** | **Diam vs LO (0.0199)**  **NPi vs LO (0.0005)**  **NPi vs Diam (0.1068)** |
| **27.5** | **-37.91**  **(-83.46, 7.64)** | **-9.57**  **(-23.98, 4.84)** | **-2.86**  **(-13.88, 8.15)** | **1.977**  **(0.3722)** | **Diam vs LO (0.2410)**  **NPi vs LO (0.0799)**  **NPi vs Diam (0.2410)** |
| **30** | **-27.99**  **(-69.90, 13.91)** | **-8.90**  **(-20.24, 2.44)** | **2.90**  **(-6.88, 8.03)** | **4.784**  **(0.0915)** | **Diam vs LO (0.3524)**  **NPi vs LO (0.0198)**  **NPi vs Diam (0.0467)** |
| **32.5** | **-29.10**  **(-59.15, 0.95)** | **-3.53**  **(-8.84, 1.77)** | **-2.81**  **(-13.28, 7.67)** | **3.310**  **(0.1911)** | **Diam vs LO (0.0882)**  **NPi vs LO (0.0418)**  **NPi vs Diam (0.3525)** |
| **35** | **-27.36**  **(-64.03, 9.30)** | **-5.64**  **(-21.60, -10.33)** | **-3.32**  **(-10.73, 4.08)** | **4.824**  **(0.0896)** | **Diam vs LO (0.0798)**  **NPi vs LO (0.0152)**  **NPi vs Diam (0.2244)** |
